# PCSK9 and the nervous system: a no-brainer?

**DOI:** 10.1016/j.jlr.2023.100426

**Published:** 2023-08-14

**Authors:** Ali K. Jaafar, Romuald Techer, Kévin Chemello, Gilles Lambert, Steeve Bourane

**Affiliations:** 1 Laboratoire Inserm UMR 1188 DéTROI, Saint-Pierre, La Réunion, France; 2 Faculté de Médecine, Université de La Réunion, Saint-Pierre, La Réunion, France

**Keywords:** PCSK9, Nervous system, Brain, Alzheimer’s disease, Stroke, LDL receptor

## Abstract

In the past 20 years, PCSK9 has been shown to play a pivotal role in LDL cholesterol metabolism and cardiovascular health by inducing the lysosomal degradation of the LDL receptor. PCSK9 was discovered by the cloning of genes up-regulated after apoptosis induced by serum deprivation in primary cerebellar neurons, but despite its initial identification in the brain, the precise role of PCSK9 in the nervous system remains to be clearly established. The present article is a comprehensive review of studies published or in print before July 2023 that have investigated the expression pattern of PCSK9, its effects on lipid metabolism as well as its putative roles specifically in the central and peripheral nervous systems, with a special focus on cerebrovascular and neurodegenerative diseases.

Proprotein convertase subtilisin/kexin type 9 (PCSK9) is a serine protease primarily expressed by liver cells (1). It is synthesized as a precursor that undergoes autocatalytic intramolecular processing to form a mature protein that is secreted. In 2003, Abifadel and colleagues identified gain-of-function mutations in the *PCSK9* gene causative of familial hypercholesterolemia, an autosomal dominant trait characterized by an elevation in circulating low-density lipoprotein cholesterol (LDL-C) and premature coronary heart disease (CHD) risk (2). In 2005, a causative association was established between the presence of loss-of-function mutations in *PCSK9* and lifelong reductions in LDL-C associated with lower CHD risk (3).

Evidence for the direct role of PCSK9 in LDL metabolism came from a series of studies showing that overexpression of PCSK9 promotes the plasmatic accumulation of LDL-C in mice. Conversely, PCSK9 knockout (KO) mice are hypocholesterolemic (4). Mechanistically, the LDLR promotes the cellular uptake of LDL particles by endocytosis. In the absence of PCSK9, the acidic environment of the endosome induces the dissociation of the receptor from the particle, and the LDLR is recycled back to the cell surface while the LDL particle is routed to the lysosome for degradation. When PCSK9 binds to the LDLR at the surface of cells, it undergoes endocytosis, and the LDLR fails to change conformation in the endosome, precluding normal recycling to the plasma membrane. The LDLR then traffics to the lysosome where it is degraded along with the LDL particle (5).

Given the mode of action of PCSK9 that acts primarily as a circulating inhibitor of the LDLR (6), as well as the healthy profile of individuals with reduced or absent PCSK9 function (7, 8) PCSK9 rapidly gained the status of a very promising drug target to lower LDL-C in humans with the ultimate goal of lowering the risk of heart disease. Several drug development strategies have been undertaken to pharmacologically inhibit PCSK9, the most advanced being two fully human monoclonal antibodies tested in large phase III outcome trials: the FOURIER program for evolocumab, and the ODYSSEY program for alirocumab (9, 10). Both have unequivocally shown that PCSK9 inhibition safely lowers LDL-C levels [56 mg/dl absolute reduction (95% confidence interval 55–57) or 59% relative reduction (95% confidence interval 58–60)] alone or on top of maximally tolerated lipid-lowering therapies (i.e. high-doses statins with or without ezetimibe) and reduces the number of cardiovascular events. In addition to monoclonal antibodies that sequester PCSK9 in the circulation and are now prescribed to patients, the small-interfering RNA inclisiran that targets PCSK9 hepatic production has recently been approved, following reports of substantial LDL lowering [50% reduction (95% confidence interval 47–53)] with this drug (11).

Beyond its central role in LDL-C regulation and cardiovascular health, distinct biological effects of PCSK9 have been reported, notably in septic shock, vascular inflammation, viral infection, and cancer (12). These novel roles have been proposed to result, at least in part, from the ability of PCSK9 to bind to and enhance the degradation of other members of the LDLR family of receptors but also of the scavenger receptor CD36, CD81, the epithelial sodium channel (ENaC), and the major histocompatibility type I receptor. Yet, the precise functions of PCSK9 in extra-hepatic tissues remain a matter of considerable debate (Fig. 1). For instance, PCSK9 is expressed in the intestine, kidney, and pancreatic beta cells, but animal models conditionally lacking PCSK9 in these organs did not successfully highlight an essential role for PCSK9 in these tissues. This is further underlined by the overt phenotype of PCSK9 liver conditional knockout mice, an animal model characterized by a total absence of circulating PCSK9 as well as a lipoprotein profile almost similar to that of complete knockouts (13). Originally known as Neural Apoptosis-Regulated Convertase 1 (NARC1), PCSK9 was discovered by the cloning of genes up-regulated after apoptosis induced by serum deprivation in primary cerebellar neurons (1), but despite its initial identification in the brain, the precise roles of PCSK9 in the central (CNS) and peripheral (PNS) nervous systems remain largely elusive.

**Fig. 1:**
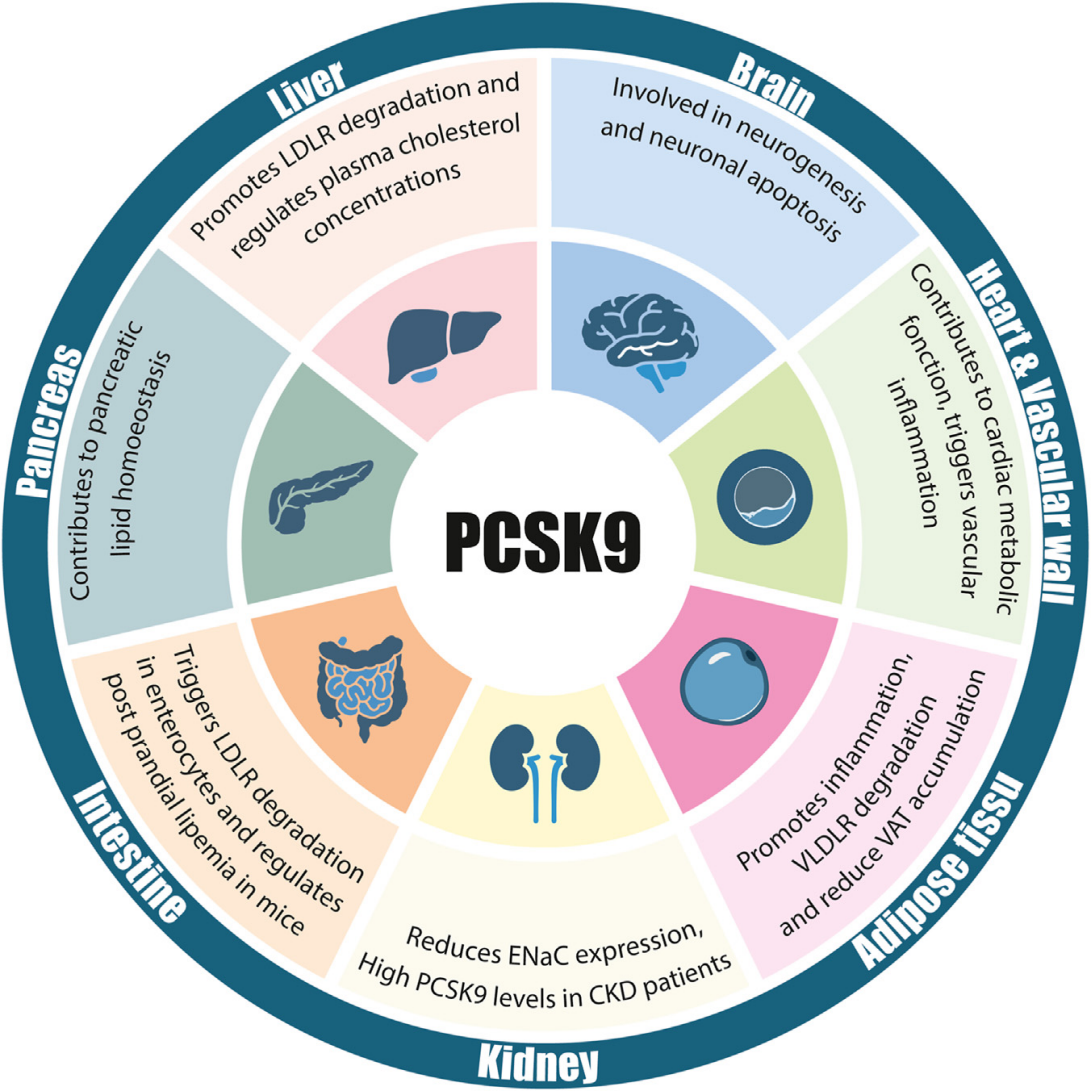
**The multifaceted roles of PCSK9 in extrahepatic tissues.** CKD, chronic kidney disease; VAT, visceral adipose tissue.

## Lipids are key components of the nervous system

The nervous system is one of the most lipid-rich tissues of the human body, and the brain is the richest in cholesterol (14, 15). Lipids account for more than a third of the dry weight of neuronal bodies, 57% being phospholipids and 15% cholesterol, (16). As key determinants of membranes, lipids largely contribute to the structural integrity and physical properties of nerve cells (17, 18, 19). Most lipids of the nervous system are localized in myelin, a specialized membrane that forms a multilayered sheath around axons exclusively in the central and peripheral nervous system. Myelin is characterized by a very high lipid/protein ratio (20, 21, 22) and is particularly enriched in cholesterol (23), with a relative proportion of 40% cholesterol, 40% phospholipids, and 20% glycolipids. The ratio is closer to 25%:65%:10% in most other biological membranes (24). Table 1 comparatively displays the lipid contents of myelin (in both the central and peripheral nervous systems) and hepatocyte membranes (25, 26, 27). Myelin’s unique lipid composition supports its critical role in the nervous system as it allows rapid saltatory conduction of nerve impulses and provides trophic support along the axon. Slight changes in the lipid composition of myelin sheath can alter adhesive properties and lead to structural disruptions (28) and serious neurological disorders (29).

**Table 1:** Lipid composition of myelin in the peripheral nervous system (PNS), central nervous system (CNS) and of liver cells plasma membranes

	PNS myelin	CNS myelin	Liver Plasma Membranes
Cholesterol	41%	46%	17%
Phospholipids	42%	33%	59%
Phosphatidylethanolamine (PE)	12%	13%	7%
Phosphatidylcholine (PC)	10%	7%	24%
Phosphatidylserine (PS)	7%	7%	4%
Phosphatidylinositol (PI)	<1%	<1%	4%
Sphingomyelin (SM)	13%	6%	20%
Glycolipids	11%	20%	7%
Cerebrosides	10%	17%	-
Sulfatide	1%	3%	-
Other lipids	6%	1%	17%

All values are expressed as molar percentages of total lipids.

In addition, lipids are involved in various signaling processes that regulate neuronal survival and differentiation (30, 31) and can also be used as energy substrates (32) or during cellular stress in the CNS (33). The molecular pathways governing lipid metabolism in the nervous system are tightly regulated, their central role in nerve function and CNS homeostasis being clearly underlined by the observation that disturbances of lipid homeostasis often associate with the onset of neurological disorders (34, 35). For instance, inherited disorders of cholesterol biosynthesis (e.g. the Smith-Lemli-Opitz syndrome) or metabolism (e.g. Cerebrotendinous Xanthomatosis) are causative of severe neurological symptoms and possibly myelin defects (36, 37). Given the lipid-demanding nature of the nervous system, and the central role of PCSK9 in cholesterol metabolism, several studies have explored the expression and function of PCSK9 in these tissues.

## A convertase implicated in neural apoptosis and neurogenesis

### Apoptosis

PCSK9 was initially discovered in apoptotic primary cerebellar neurons (1, 38). PCSK9 exhibits pro-apoptotic properties in potassium-deprived cerebellar granule neurons and nerve growth factor-deprived dorsal root ganglion neurons, by reducing apolipoprotein E receptor 2 (ApoER2) abundance and thereby anti-apoptotic signaling pathways (39, 40). Likewise, PCSK9 inhibition attenuates neuronal apoptosis after middle cerebral artery occlusion (MCAO) injury in hyperlipidemic mice, an effect also associated with apoER2 upregulation (41). In contrast, PCSK9 displays anti-apoptotic properties in U251 human glioma cells. PCSK9 gene silencing leads to cell shrinkage, loss of membrane integrity, nuclear fragmentation, and chromatin compaction, whereas PCSK9 overexpression in these glioma cells restores normal morphology (42). Thus, it seems that PCSK9 could have both pro- and anti-apoptotic effects in different cell lines. In vivo, data about the role of PCSK9 in apoptosis are still very limited and need further investigation to confirm these observations.

### Neurogenesis

Given the critical role of apoptosis during the development or degeneration phases of the nervous system, studies have been undertaken to unravel the role of PCSK9 in neurogenesis. PCSK9 expression is detectable during the early stages of neurogenesis (three somite stages, 10.33 h post fertilization in zebrafish) and during telencephalic and cerebellar embryonic neurogenesis (E12.5 and E17-P15, respectively, in mice). PCSK9 expression in the adult brain is low and only observed in areas where neurogenesis occurs, such as cortical, intracranial, and cerebellar granule neurons in zebrafish and the rostral extension of the olfactory peduncle (RE-OP) in mice (1, 43, 44). In humans, PCSK9 can be detected in the cerebrospinal fluid, albeit at much lower concentrations than in the plasma (on average 5 and 200 ng/ml, respectively) (45). PCSK9 overexpression in mouse embryonic neural progenitor cells leads to an increase in the number of postmitotic neurons from undifferentiated neuroepithelial cells (1). The role of PCSK9 in neuronal differentiation does not seem to be mediated by the LDLR, since neuroectodermal induction with retinoic acid of P19 mouse embryonic carcinoma cells increases endogenous PCSK9 mRNA expression, without altering LDLR protein abundance nor Sterol Regulatory Element Binding Protein 2 mRNA expression levels (43). Knocking-down PCSK9 is lethal in zebrafish, as a result of defective neurogenesis and total absence of midbrain-hindbrain boundary (43). However, PCSK9 is not necessary for survival in mammals raising the question about a compensatory action of other factors during evolution.

## A regulator for the brain

**Fig. 2:**
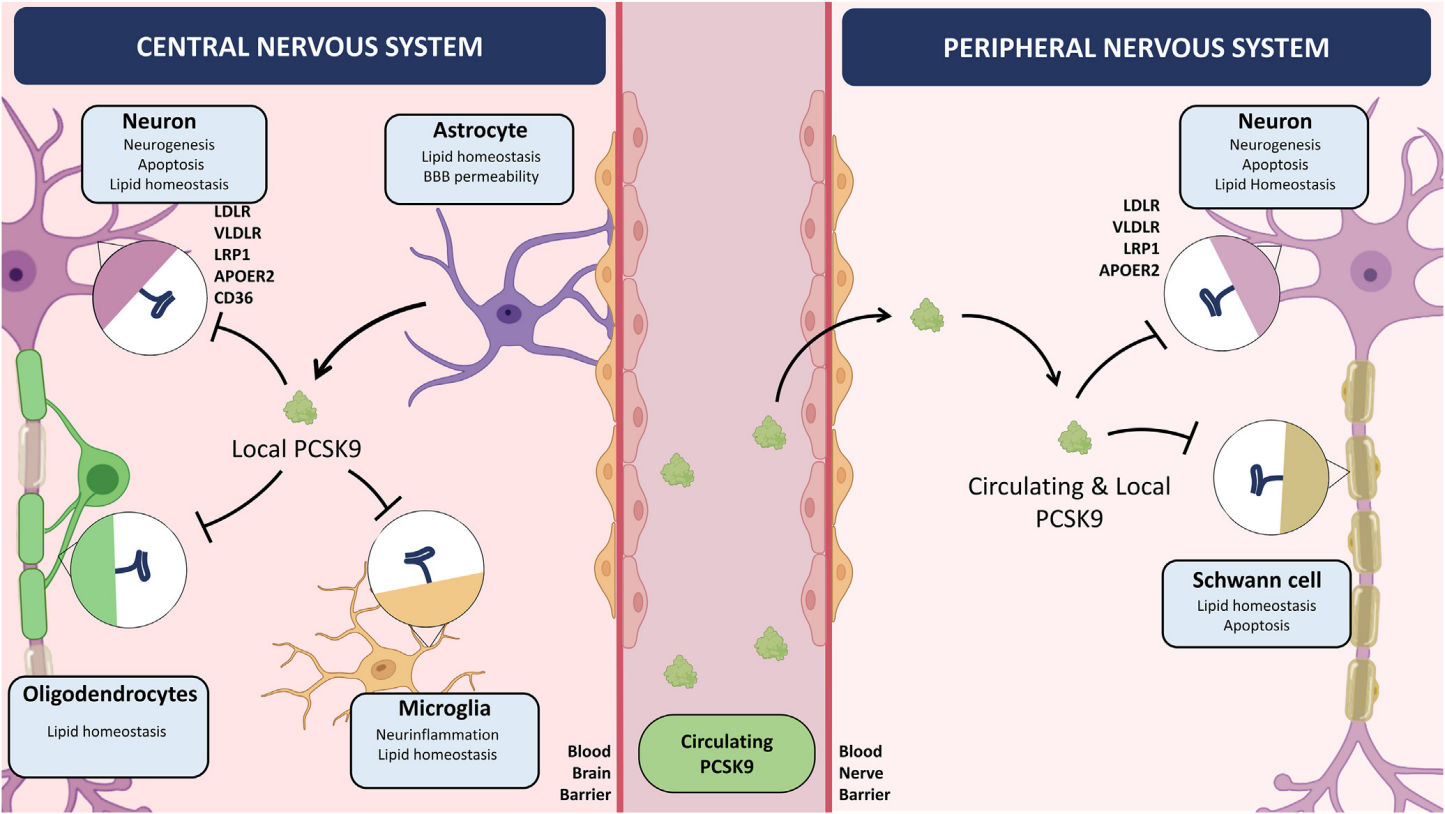
**Proposed roles for PCSK9 in the central and peripheral nervous systems.** In the CNS, circulating PCSK9 only directly interacts with vascular endothelial cells and astrocytes as it does not cross the BBB in normal conditions. In contrast, PCSK9 produced locally may act on LDLR, LRP1, VLDLR, and apoER2 are expressed by neurons, astrocytes, and oligodendrocytes as well as on LRP1 and CD36 that are expressed by microglia. These receptors are known to modulate lipid homeostasis, neurogenesis, apoptosis, and inflammatory processes in the brain. In the PNS, both circulating and locally produced PCSK9 have the ability to modulate LDLR, LRP1, ApoER2, and VLDLR that are expressed primarily by neurons and Schwann cells and thereby regulate lipid homeostasis and apoptotic processes in peripheral nerves.

In the brain, PCSK9 potentially interacts with several members of the LDLR family of receptors that transport cholesterol into neurons (i.e..LDLR, the very-low-density lipoprotein receptor (VLDLR), ApoER2, and the LDLR-related protein 1 (LRP1)) as well as with CD36 that transports fatty acids (12). In vitro and in vivo studies conducted in mice provide conflicting evidence on whether PCSK9 targets these receptors for degradation. Since neither plasma lipoproteins nor circulating PCSK9 have the ability to cross the blood–brain barrier (BBB) under physiological circumstances (45, 46, 47), only locally expressed PCSK9 may directly modulate cholesterol homeostasis in the developing brain by promoting lysosomal degradation of these receptors (Fig. 2).

LDLR family members have a high affinity for apolipoprotein E (apoE), a major carrier of cholesterol in the brain, allowing transport of apoE-associated cholesterol from pericellular fluids into neurons (44). In vitro, PCSK9 was shown to reduce LDLR abundance in neurons (48). In vivo, LDLR protein levels are significantly higher in the telencephalon and cerebellum of PCSK9 knockout mouse embryos, and levels of non-truncated apoE are reduced, compared with wild types (44). However, in adult animals, despite the co-localization of PCSK9 and LDLR transcripts in RE-OP, PCSK9 KO mice do not display significantly different levels of LDLR and apoE proteins in their olfactory bulb or cerebrospinal fluid compared with wild-types (44). Likewise, overexpression or ablation of PCSK9 did not alter LDLR, VLDLR, or ApoER2 expression levels in the hippocampus and cerebral cortex of adult mice (49), nor did they alter the cortical development of the animals (48). Even if knocking-out PCSK9 were to modulate the relative abundance of several lipid species in the cortex and cerebellum of adult mice, these animals do not show any sign of behavioral abnormality (48). In contrast, PCSK9 concentrations are lower in the plasma and amniotic fluid of pregnant female rats as well as in rat embryos with experimentally induced neural tube defects (50) and the plasma of pregnant women with fetuses suffering from open neural tube defects (51).

The adult brain vastly relies on endogenous synthesis for its cholesterol needs since the BBB is virtually impermeable to circulating lipoproteins (52) and since, unlike oxysterols, cholesterol molecules do not cross the BBB (53). Cells of the CNS, including neurons, synthesize cholesterol. However, adult neurons gradually lose this ability and become dependent on cholesterol supplied by astrocytes (15, 54, 55). Lipid homeostasis in astrocytes is central to brain health. Thus, recent studies have shown that abnormal lipid accumulation in astrocytes can lead to the formation of lipid-accumulated reactive astrocytes, promoting recurrent neuronal firing and ultimately leading to neuronal loss (56). Lipoproteins and their receptors play important roles in the regulation of neurobehavioral function and energy balance in the brain (57). Brain lipid homeostasis is thus subject, to some extent, to the action of local and/or circulating PCSK9 on neurons and astrocytes that are in direct contact with the cerebrovascular endothelium.

## PCSK9 in the peripheral nervous system

To date, the expression of PCSK9 in the peripheral nervous system is not firmly established. The only piece of evidence of PCSK9 expression in peripheral nerves is the observation that PCSK9 transcripts are expressed in a rat Schwann cell line (1). Given that some neurons of the CNS express PCSK9, it cannot be ruled out that PCSK9 is also produced in neural and/or glial cells of the PNS. According to the Genotype-Tissue Expression (GTEx) portal, PCSK9 is synthesized in human tibial nerves at levels deemed physiologically relevant (58). PCSK9 could potentially impact lipid metabolism locally in the peripheral nervous system and thus modulate peripheral nerve physiology.

A distinct feature of peripheral nerves is that they are located outside of the BBB. The blood–nerve endothelial barrier has distinct permeability characteristics (59) making peripheral nerves more susceptible to the action of systemic PCSK9 and hence to PCSK9 inhibitor treatments (Fig. 2). Incidentally, a recent case has reported a patient with a history of prediabetes and dyslipidemia treated with PCSK9 inhibitors who developed peripheral neuropathy (60). The patient started evolocumab treatment due to statin intolerance. While on PCSK9 inhibitor, the patient developed symptoms of chronic inflammatory demyelinating polyneuropathy characterized by bilateral thigh pain, weakness, and numbness. The symptoms improved after stopping evolocumab but resumed a few weeks later after the patient had been injected with the other PCSK9 inhibitor, alirocumab. There is no other report of an association between PCSK9 inhibitor use and demyelinating polyneuropathies. Of note, even if several cases of statin-induced peripheral neuropathy have been reported, there is no evidence for a causal relationship between statin treatment and peripheral neuropathy (61). In addition, a positive association was found between corneal nerve loss and circulating PCSK9 (62), and PCSK9 inhibitors treatment resulted in fibers regeneration in familial hypercholesterolemic patients with corneal small nerve fibers degeneration (63). Randomized trials with these drugs do not show any significant musculoskeletal and neurodegenerative side effect (64), even if several members of the LDLR family of receptors are duly expressed in peripheral nerves and mediate important interactions between Schwann cells and axons as well as Schwann cell response to injury in vivo. (65, 66).

Peripheral axons are more vulnerable compared to the brain due to their polarity, length, and the absence of an impermeable blood–nerve barrier. Schwann cells and the lipid-rich myelin sheath are essential for adequate homeostasis of peripheral nerves. It is therefore likely that PCSK9 could play a role in the regulation of lipid metabolism in the PNS in normal or pathological conditions. The importance of lipoprotein uptake for the maintenance of PNS lipid homeostasis is further highlighted by the abundance of lipoprotein receptors in peripheral nerves. Schwann cells express LDLR (67), LRP-1 (66), and ApoER2 (65). These receptors have also been identified in the dorsal root ganglia along with the VLDLR (68) (Fig. 2).

## PCSK9 and stroke

An obvious detrimental role for PCSK9 on brain health is by increasing LDL-C levels which accelerates intracranial atherosclerosis (in particular of large vessels) leading to ischemic strokes. This was evidenced in carriers of PCSK9 gain-of-function mutations (69, 70). In line with these observations, PCSK9 inhibition with evolocumab reduced ischemic strokes and major cardiovascular events in the total population of the FOURIER trial as well as in the key subgroup of patients with prior ischemic strokes (71). Whereas PCSK9 loss-of-function mutations carriers with lifelong reductions in LDL-C and coronary heart disease incidence, were not initially found at significantly reduced risk of stroke (3, 72), a much larger study of the UK Biobank database described the first genetic evidence of a protective effect of the R46L loss-of-function mutation on PCSK9 on ischemic stroke (73).

In contrast to ischemic strokes, only one study has investigated a link between PCSK9 and brain hemorrhage, showing no significant association (73). Nevertheless, PCSK9 inhibition has been proposed to precipitate hemorrhagic cerebral infarcts and to associate with increased cerebrovascular-related mortality (72, 74, 75), as a result of very low LDL-C levels, reduced platelets reactivity (via CD36), and lower blood coagulability (via LRP1) (76, 77). *In vivo* studies designed to investigate this possibility did not show any difference between PCSK9 knockout and wild-type mice in terms of cerebral infarct size following a transient MCAO procedure. PCSK9 KO and control mice also displayed similar hemorrhagic transformations following reperfusion post-MCAO (44, 74). These studies were however performed in young and otherwise healthy animals which do not recapitulate the pathophysiological conditions of ischemic strokes (aging population, diabetes, and hypertension). In that respect, aging PCSK9 KO mice accumulate cholesterol in pancreatic islets and display impaired glucose-stimulated insulin secretion irrespective of gender and diet as well as signs of glucose intolerance (78). Given that hyperglycemia and diabetes alone worsen ischemic strokes outcome in terms of prevalence, severity, and complications, and negatively impact on the integrity of the BBB, the initial hypothesis has been revisited: under acute hyperglycemic conditions, PCSK9 KO mice exhibit more intracerebral hemorrhages than wild-type mice following an MCAO procedure (79). Likewise, compared with placebo, PCSK9 inhibition with monoclonal antibodies increased cerebral hematoma volume following induction of experimental intracerebral hemorrhage using collagenase in a mouse model harboring a lipoprotein profile similar to that of humans (80). Lowering PCSK9 and LDL-C therefore appears beneficial in the context of ischemic stroke but further investigations are needed to test whether PCSK9 inhibition impacts hemorrhagic stroke.

## PCSK9 and neurocognition

Impairment of neurocognitive function has been an important concern for the clinical development of PCSK9 inhibitors. In the initial trials of these drugs, the incidence of neurocognitive adverse events (including delirium, cognitive and attentional disturbances, dementia, thought and perceptual disturbances, and psychiatric disorders) was very low (<1%) but slightly higher albeit non-significantly in the treated groups compared with placebo (0.9% vs. 0.3% for evolocumab) (81) (1.2% vs. 0.5% for alirocumab) (82). However, the combined analysis of both studies indicated an increased occurrence of neurocognitive side effects in patients treated with PCSK9 inhibitors (83). But the analysis of larger groups of patients enrolled in those trials over longer follow-up periods did not show significantly more neurocognitive adverse events associated with these therapies (10, 84, 85). Furthermore, there was absolutely no correlation between the occurrence of neurocognitive side effects and the degree of LDL-C reduction achieved with these drugs (64).

Likewise, studies conducted in PCSK9 loss-of-function mutations carriers did not show any sign of neurocognitive impairment or decline over time (86), indicating that lifetime exposure to low levels of PCSK9 and thus of LDL-C does not impact neurocognitive function. Of note, PCSK9 loss-of-function mutations were found associated with depression in Mendelian randomized analyses using the British Biobank cohort (87) and in a genome-wide association study (88).

## PCSK9 and Alzheimer’s disease

Considering that PCSK9 was initially found in apoptotic neurons, its implication in neurodegenerative diseases, in particular in Alzheimer’s disease (AD) has been extensively scrutinized (1). AD is characterized by the accumulation in the brain of toxic β-amyloid peptides arising from the cleavage of amyloid precursor proteins by β-site amyloid precursor protein cleaving enzyme (BACE1). *In vitro*, PCSK9 overexpression reduced BACE1 expression whereas PCSK9 inhibition increased the levels of BACE1 and Aβ deposition (89). A series of experiments conducted in rat stroke models found a negative association between the expression of PCSK9 and the formation of amyloid plaques (90). However, another study failed to identify a direct effect of PCSK9 on BACE1 expression or Aβ levels in mice (49).

A second putative pathway that may link PCSK9 inhibition to AD includes its inhibitory effects on the LDLR family of receptors as well as on the scavenger receptor CD36 in the central nervous system (91). Thus, PCSK9 inhibits the expression of the LRP8 (also known as apoER2), a receptor involved in signaling pathways governing the survival of neurons (39). In addition, genetic variants in the *LRP8* locus associate with an increased risk of AD (92). Likewise, two other PCSK9 targets, LRP1 and CD36, have been proposed to play a role in Aβ clearance (91). Thus, endothelial expression of LRP1 promotes the clearance of Aβ across the BBB in vivo, a process directly modulated by PCSK9 inhibitor treatment (93, 94), and the expression of CD36 parallels the ability of microglia to perform Aβ clearance (95). The ability of PCSK9 to modulate the abundance of these receptors in the CNS is still unknown. Another mechanistic pathway linking PCSK9 to AD, as shown in vitro, would be through canonical inhibition of local LDLR expression, resulting in decreased astrocyte-to-neuron cholesterol transport and reduced neuronal cholesterol content, thereby increasing Aβ neurotoxicity (96). Presumably, by reducing LDL-C, PCSK9 inhibitors may also lower the inflammatory processes within cerebral blood vessels and thus mitigate the progression of AD (97).

In that respect, PCSK9 concentrations were found higher in the cerebrospinal fluid as well as brain autopsies of AD patients compared with controls (98), but similar observations have been made for other neurodegenerative diseases (99). Noteworthy, there is no consensus for an association between PCSK9 genetic variants and AD, given that PCSK9 loss of function mutations carriers are at neutral risk of developing AD (86, 100, 101).

The impact of PCSK9 in other neurodegenerative diseases such as Parkinson’s disease, amyotrophic lateral sclerosis, multiple sclerosis, and Huntington’s disease with severe neuronal apoptosis has not been investigated despite the recognized association between lipid metabolism and the severity of these diseases (102, 103, 104, 105). In a mouse model of multiple sclerosis, PCSK9 did not alter the progression of the disease or the associated immune responses (106). Yet, more work is needed to assess any potential impact of PCSK9 on these pathologies.

## Conclusion

The best-characterized function of PCSK9 is its inhibitory action on hepatocyte-derived LDLR and thereby its established role in the systemic control of blood cholesterol. Despite its initial identification in the brain, the precise roles of PCSK9 in the nervous system remain elusive. In vitro, in vivo, epidemiological, and genetic studies indicate that PCSK9 possesses neurobiological regulatory properties of relevance in pathophysiological conditions of the nervous system. If so, it remains to be established whether these disorders are influenced by PCSK9 expressed locally in nervous tissues or systemically by circulating PCSK9. It is also important to decipher the precise molecular and cellular pathways acted upon by PCSK9 in these tissues. Important efforts should therefore be deployed to unravel the yet unknown functions of PCSK9 in the central and peripheral nervous systems. PCSK9, the youngest member of the family of proprotein convertases, has made history in cardiology but still raises many hopes of new clinical applications beyond cardiovascular health.

## Conflict of interest

The authors declare that they have no known competing financial interests or personal relationships that could have appeared to influence the work reported in this paper.
